# The barriers and facilitators to the implementation of National Clinical Programmes in Ireland: using the MRC framework for process evaluations

**DOI:** 10.1186/s12913-018-3543-6

**Published:** 2018-09-24

**Authors:** Catherine D. Darker, Gail H. Nicolson, Aine Carroll, Joe M. Barry

**Affiliations:** 1 Department of Public Health & Primary Care, Institute of Population Health, School of Medicine, Trinity College Dublin, Russell Centre, Tallaght Cross, Dublin, D24 DH74 Ireland; 2 Clinical Strategy and Programmes Division, Health Service Executive, Dr Steevens’ Hospital, Steeven’s Lane, Dublin, 8, D08 W2A8 Ireland; 3 School of Medicine, University College Dublin, Dublin, Ireland

**Keywords:** Healthcare, Implementation, Process evaluation, MRC framework, Qualitative

## Abstract

**Background:**

A major healthcare reform agenda in Ireland is underway which underpins the establishment of a series of National Clinical Programmes (NCPs), which aim to take an evidence based approach to improve quality, access and value. The current study aimed to determine the enablers and barriers to implementation of the NCPs.

**Methods:**

A qualitative methodology advocated by the Medical Research Council (MRC) framework on conducting process evaluations of complex interventions guided this research. Purposive sampling techniques were used to recruit participants from seven NCPs across both acute and chronic healthcare domains, comprised of orthopaedics, rheumatology, elective surgery, emergency medicine, paediatrics, diabetes and chronic obstructive pulmonary disease. A total of 33 participants were interviewed using a semi-structured interview guide. Participants included current and previous Clinical Leads, Programme Managers, Health Service Executive management, hospital Chief Executive Officers, representatives of General Practice, and a Nursing and a Patient representative. Thematic analyses was conducted.

**Results:**

A range of factors of different combinations and co-occurrence were highlighted across a total of six themes, including (i) positive leadership, governance and clinical networks of the NCPs, (ii) the political and social context in which the NCPs operate, (iii) constraints on resources, (iv) a passive attitudinal resistance to change borne from poor consultation and communication, (v) lack of data and information technology, (vi) forces outside of the NCPs such as the general practitioner contract thwarting change of the model of care.

**Conclusions:**

The MRC framework proved a useful tool to conduct this process evaluation. Results from this research provide real world experiences and insight from the people charged with implementing large-scale health system improvement initiatives. The findings highlight the need for measured responses that acknowledge both direct and non-direct challenges and opportunities for successful change. Combined, it is recommended that these elements be considered in the planning and implementation of large-scale initiatives across healthcare delivery systems, both in Ireland and internationally.

## Background

Innovations within a healthcare system are considered a driving force to improve quality, reduce harm, improve access, increase efficiency, eliminate waste, and lower costs, [1]. However, healthcare systems are faced with major challenges in the sustainability and upscaling of innovations [2, 3]. It is insufficient to improve the performance of the health service by addressing interventions at local level and individual practitioner behaviours only [4]. What is required to provide an innovation ‘at scale’ is a responsive health system which is effective, efficient and has engagement at all levels [5, 6]. These ‘large-system transformations’ [7] can only be achieved by addressing the range of complexities that can co-exist within any health system such as culture and climate, organisational governance, financial systems, health technologies, and workforce attitudes and behaviours [4].

Health service interventions are often highly complex and comprise multiple interacting components [8]. Added to this complexity includes the challenges of innovation implementation and the targeting of multiple organisational levels at any one time [9]. This complexity can present a challenge to evaluation. High quality evaluation is crucial to allow policy makers, practitioners and researchers to identify interventions that are effective, and learn how to address problems within interventions that are floundering. The United Kingdom’s Medical Research Council (MRC) identifies the value in conducting a process evaluation which can play an important role in considering the feasibility of an innovative intervention and suggesting ways in which to optimise its design, delivery and mechanisms of impact [10]. The MRC process evaluation guidance can assist in the understanding of the causal assumptions which underpin the intervention, and how the interventions work in a real-world context. This in turn can be helpful in building an evidence base for practice and policy.

As part of a major healthcare provision reform agenda in Ireland in 2009 the Health Service Executive (HSE) established the Quality and Clinical Care Directorate, which was subsequently divided into the Quality and Patient Safety Directorate and the Clinical Strategy and Programmes Directorate (CSPD), which became Divisions when the new Health Services Directorate was established [11]. The CSPD’s role was to develop a national, strategic and co-ordinated approach for the design of clinical service improvements, in order to deliver the triple aim [12] of improved patient care, improved access, and better use of resources. The National Clinical Programmes (NCPs) were established in 2010 as key drivers of this change, and were established jointly by the HSE CSPD, and the Forum of Post Graduate Training Bodies, such as the Royal College of Physicians of Ireland [13]. The NCPs are at different stages of evolution with some Programmes being in the advanced stage of implementation and some Programmes still being in the design stage. Each of the NCPs has a Clinical Lead, a multi-disciplinary Working Group (including patient representatives), and a Clinical Advisory Group. This brings together clinical and management healthcare professionals across relevant disciplines into a clinical network to design and specify standardised models of care, guidelines, pathways and associated strategies for the delivery of clinical care. The establishment of the NCPs is a large-scale ‘systems level’ intervention within the Irish healthcare service, which has networks as a core component.

The aim of the current study was to develop a detailed understanding of the implementation of the NCPs and all of its complexity. Particular emphasises was placed on facilitative factors and barriers. This will yield important insights into what is required for overall implementation success, and will provide valuable information for future planning and optimal development of programmes success.

## Methods

### Design

The design of this study is qualitative in nature. Stakeholder interviews are a common method of process evaluation inquiry as outlined by the MRC framework to ‘capture emerging changes in implementation, experiences of the intervention and unanticipated or complex causal pathways’ [10]. For the first time since the NCPs’ inception, in-depth qualitative data was collected to provide a detailed understanding of programme implementation, and barriers and enablers arising from same. The evaluation approach is inductive to the extent that researchers attempted to make sense of the situation without imposing pre-existing expectations on the setting under inquiry, and holistic in the sense that it was assumed that the whole is understood as a complex system that is greater than the sum of its parts [14].

### Selection of National Clinical Programmes and participants

At the time of conducting this study, there were a total of 33 NCPs. A subset of these were purposively selected with the objective of including programmes from across both acute and chronic healthcare domains. A purposive sample is a non-probability sample that is selected based on characteristics of a participant, such as knowledge and expertise, and the objective of the study [15]. The current study focused on seven NCPs and comprised of orthopaedics, rheumatology, elective surgery, emergency medicine, paediatrics, diabetes and chronic obstructive pulmonary disease selected to cover the diversity of the NCPs on the basis of cross specialty (medicine and surgery), disease specific versus system (e.g. diabetes versus paediatrics) and differing stages of evolution (orthopaedics, in the advanced stages of implementation and COPD in the design stages).

The sampling procedure was targeted specifically at key stakeholders involved in the NCPs. A total of 33 participants (comprising 22 males, 11 females) were interviewed. Participants included ten current and previous Clinical Leads, eight Programme Managers, nine members of Health Service Executive management, two hospital Chief Executive Officers, two representatives of General Practitioners and one of Nursing, and a Patient advocate. In essence, purposive sampling, by its nature is a non-representative subset of some larger population, and is constructed to serve a very specific need or purpose. The objective of this study sought to gain insight and experience from a range of knowledgeable experts across various and different levels of involvement, such as at each individual NCP level, as well as at systems- and organisation level, and from a patient perspective. Some of the Clinical Leads and Programme Managers fulfilled their role for more than one NCP at a time, or were either currently or previously in the role, so were therefore in a position to help build temporal layers of knowledge and insight of the NCPs. The focus of recruitment was to choose information-rich cases from which it was possible to learn a great deal about issues of central importance to the purpose of the inquiry, i.e. to identify the barriers and enablers to implementation of the NCPs.

### Procedure

Participants were invited to participate through a personally addressed email sent from the National Director of the CSPD. A semi-structured interview schedule guided the interviews which allowed for probing, follow-up questions and flexibility about a particular topic or theme that emerged [16]. The interview schedule was piloted to inform relevant modifications and for ease of administration. Interview questions were derived through consultation with the CSPD Directorate, a thorough literature review and discussions within the research team. (See Appendix for sample interview schedule). All of the interviews were conducted face-to-face and took place between November 2016 and March 2017. Interviews were carried out at a time and location convenient to participants. Only the interviewer (GN) and interviewees were present at time of interview. All interviews were audio-recorded. Each interview lasted on average one hour. A professional transcriber, who signed a confidentiality agreement, transcribed the recordings verbatim. Field notes were completed after each interview.

Informed written consent was obtained prior to commencing interviews. Process evaluations typically involve collecting rich data from a limited pool of participants, and the issue of confidentiality is key [10]. With this in mind, care was given to anonymise any identifiable information. Confidentiality was assured and participants were advised that they would not be identified by name but rather by their role with regard to the NCPs. Therefore, in reporting the results, participants are referred to as ‘Clinical Lead 1’, ‘GP Representative 1’ and so on. The study methods followed published standards for undertaking and reporting qualitative research (COREQ) [17]. Ethical approval was obtained from the Research Ethics Committee of the School of Medicine, Trinity College Dublin (reference: 20160506).

### Data analysis

Thematic analysis was used as the analytical method. It is a method for identifying, analysing, organising, describing, and reporting themes found within qualitative data [18]. Thematic analysis provides a highly flexible approach, providing a rich and detailed, yet complex account of data [19]. The process of coding used in the current study was drawn from the process of coding in six phases: familiarisation with data, generating initial codes, searching for themes among codes, reviewing themes, defining and naming themes, and producing the final analyses [19]. Analyses was completed by hand and no software package was used.

Two researchers (CD & GN) independently read the transcripts. Rigorous line-by–line coding was applied, with a focus on experiential claims and concerns [20]. Patterns in the data were clustered into a thematic structure to identify and categorise major themes and sub-themes. Themes were identified when they emerged consistently in a number of transcripts. Data saturation was achieved as conceptualised by inductive thematic saturation within the analyses, in relation to the (non-)emergence of new codes or themes [21]. Themes and sub-themes were reviewed and refined to ensure they formed a coherent pattern and to recode if necessary. Any differences in interpretation were resolved through discussion. A third researcher (JB) reviewed the coding frame and applied it to a subset of four (approximately 10%) of the transcripts. This type of analytical triangulation [22] aims to reduce bias and ascertains the validity of the coding frame as an analytical tool. The kappa coefficient was calculated as 0.77, which indicated a good rate of inter-rater reliability.

## Results

In keeping with the aim of the study the results presented focus on the ‘implementation’ element of the MRC’s process evaluation framework [10], in particular the barriers and facilitators to implementation of the NCP’s. These six themes identified through interviews were: leadership, governance and clinical networks; social and political context; resources (both in terms of funding and manpower); resistance to change; data and information systems; and changing the model of care.

### Leadership, governance and clinical networks

All participants cited the importance of the role of Clinical Lead as being the “*biggest facilitating factor”* [Patient Representative] for change. Effective Clinical Leads, despite a considerable workload, were dedicated, energetic and enthusiastic overall. The importance of leadership to facilitate “*multi-disciplinary interventions”* [Clinical Lead 5], and organisational change and quality improvement was widely recognised. Respondents reported that a Clinical Lead should have sufficient high status within their discipline to be a credible source of information, to have subject matter expertise, and be a respected representative of their peers. *“The reason I’m the national Clinical Lead is because I was elected to be President of the [removed to protect identity]. I can go to [HSE CEO] and say I represent my speciality in the country and I think that’s quite a powerful thing. It also helps when I have to go back and talk with the people in my own specialty”* [Clinical Lead 5]. The role of the Programme Manager was also cited as being a hugely important supportive factor in the NCPs’ success, with many participants viewing the role of the Programme Manager as a leadership role as well: *“A key lesson was that the Programme Managers were fantastic, they worked every hour that God gave them because they were managing four or five Programmes. They had the trust of key people, and trust is vital in getting anything done”* [HSE Manager 3]. The concept of clinical networks, bringing together clinical and management healthcare professionals, with patient representation was also cited as a major facilitating factor: *“To be honest, it works well because all of the stakeholders are together, we all have a place at the table. We have everyone there – the senior Consultants, nurses, management, and we also have a patient too. We are planning, designing, making decisions, all together. That is very rare. And I can’t help but feel that is why it works so well. Everyone is ‘inside the tent’.”* [Clinical Lead 7].

Relationships between the HSE and clinicians were described as negative at the time of the NCPs establishment. In order to overcome this, the medical training Colleges were asked to be involved in the nomination process of the Clinical Leads, and in establishing Clinical Advisory Groups. This proved beneficial in the early implementation stage, as clinicians had a positive relationship with Colleges and this facilitated buy-in by clinicians to the NCPs. *“The HSE also had very little credibility at the time with clinicians so working with the College meant that the College was involved with the nomination process so identifying the Clinical Lead and nominating somebody to the HSE for appointment as the Clinical Lead”* [HSE Manager 1].

### Social and political context

Participants described the dominant, powerful and sometimes obstructive role of the wider political context, within the context of health system reform in general, and the implementation of the NCPs in particular. The Minister for Health, an elected official, was viewed as a critically important agent in the health policy decision-making process. The political cycle in Ireland is a five-year long cycle, or less should a particular crisis result in a change in Government or a change in Minister. This change in policy and political leadership can be disruptive and was noted as a barrier to the implementation of the NCPs with “*people who work in the health service suffer[ing] this learning process time and time again*” [Clinical Lead 2] where the new Minister must learn the functioning of the health system.

The interlinked factors of the media’s influence, the opinion of the general public, and the priorities of Government, had a direct impact on the decision-making process at operational level within the NCPs. “*Governments fell and there was a campaign, quite bitter, public campaign about re-opening an emergency department that wasn’t sustainable”* [Hospital CEO 2]. In Ireland, policy-makers face constant pressures from the public and media regarding numbers of patients waiting on trolleys in emergency departments, and the lengthy waiting lists for those who need outpatient treatment. This has long been a key driver for the locus of demand in the Irish health system. Although the original ambition of the NCPs was the ‘triple aim’ of quality, access and value, a tangible effort to reduce waiting lists and the numbers of patients on trolleys was imposed on the NCPs. This was cited as a pre-requisite of the NCPs before Ministerial support could be secured. *“So in the beginning [the CSPD Director] went to the Minister to explain what we wanted to do. And the Minister said ‘I completely understand what you are trying to do, you are trying to tackle chronic diseases, 80% etc. but I get beaten up about trolleys and waiting lists and unless you are doing something to tackle those I am not going to consider you a priority”* [Programme Manager 1]. However, political pressure was also seen as a positive driving force for implementation to “*push through”* [GP Representative 2] an agenda or concept to fruition, where otherwise initiatives within the NCPs would have *“reached a complete impasse*” [GP Representative 2].

### Resources

A deep recession was occurring in Ireland during the time of the initiation of the NCPs, and public expenditure in health dropped significantly [23]. This factor was outside of the control of the NCPs but deeply affected their initial implementation. “*We linked the Programmes to funding at a time of economic crisis and that absolutely hung us”* [Programme Manager 1]. However, not all participants cited a lack of funding as a major barrier to the advancement of the NCPs. Participants reflected that during the years where Ireland’s economy was thriving and financial resources were directed into the health system “*it didn’t have the outcomes that people expected”* [HSE Manager 4]*.* There was an appreciation that NCPs achieved a lot in a context of a severe economic recession and that *“they’ve also operated for the last 8 years in the most bar none, the most difficult financial environment that any advanced health system has ever been asked to work in”* [Clinical Lead 7]*.*

Participants spoke of health system financing in realistic terms, rather than thinking in terms of continuous and exponential funding. “*You could throw all the money you want at them (the NCPs), the money will run out again in a system that’s particularly ineffective and inefficient, you can keep shovelling money at it but it’s not going to make any difference”* [HSE Manager 4]. Funding that follows *“how the patient flows between the services”* [Programme Manager 2] was described as a solution to the growing costs of the health service generally, and a way to put in place a sustainable funding structure for the implementation of the NCPs. Many participants indicated that activity-based funding would be a more sensible and *“absolutely key successful funding structure”* [Clinical Lead 9] as opposed to the traditional block-based funding that has been in place to date. A core goal of the NCPs was to deliver financial value, however, participants noted that if the current block-based funding structure should prevail then the funding model “*will perversely incentivise admission over discharge”* [Clinical Lead 9], thus mitigating against the delivery of value.

Participants recognised that workforce and man-power planning were an important consideration and spoke of this in terms of implementation of the operational aspects of the NCPs. Key allied healthcare professionals, particularly nurses, were cited as a group that required additional and sustained increases in capacity, as “*we can’t recruit theatre nurses for love nor money at the moment”* [Programme Manager 2]*.* It was also understood that there was a lack of suitable potential senior personnel for the role of Clinical Leads as there “*aren’t large numbers of experienced clinicians who can deliver these Programmes walking up and down the street waiting for someone to call on them”* [HSE Manager 5]*.* Difficulties surrounding consultant recruitment was, therefore, one of the areas noted as a significant barrier to implementation.

### Resistance to change

Participants noted that some people were resistant to the introduction of the NCPs, “*there is a mixture of people wanting it to fail and inability to change”* [Clinical Lead 8], and that this resistance to change was attitudinal in nature and not necessarily as a result of other constraints as described, “*no amount of resources and funding are going to sort that basic problem”* [Patient Representative]*.* A more ‘*passive resistance’* to change was encapsulated by one respondent: “*I could break that down locally, regionally, nationally. I think the Programmes can fail locally because locally wasn’t consulted in the first place. Locally didn’t say this is actually an important area…..it was never on their agenda in the first place”* [HSE Manager 9]*.*

Communication failures in turn can cause change failures and “*there has been a lack of communication from centralised CSPD to the Programmes about what is happening”* [Clinical Lead 8]. The majority of participants in the study stated that management in the HSE ‘under-communicate’ with regard to the future direction of the NCPs. This lack of effective communication resulted in confusion and uncertainty regarding where particular roles sat within the structure of the NCPs, with one respondent commenting that Leads have said “*I’m not aligned to any of them. They’d say ‘we’ve never been told, we’ve just been told we’re doing it, we were never told we were aligned to that Programme”* [HSE Manager 3]*.* This lack of communication also extended to issues relating to executive authority to make and implement decisions. *“There’s a big gap still on who is accountable and responsible for implementation of those models of care. I still don’t know who is in charge of implementation”* [GP Representative 1]*.* Respondents made it clear that role clarity and executive authority to expedite a rapid and coordinated organisational change is essential for the future of the NCPs. This is highlighted in the following: *“It can feel at times like a little bit thankless when it comes to doing a divisional plan and you’ve done all this implementation work and a national division says to you ‘implementation isn’t your job d’you know’. That’s what I was told recently by one of the Divisions, don’t you know we implement, you don’t implement and I’m going well ‘what have I been doing for two years?’ (laughs)”* [Programme Manager 7]. Processes for facilitating effective communication and knowledge transfer were cited by participants as fundamental to the future direction of the NCPs. Communication of the NCPs’ aims and objectives was felt to be the responsibility of the individual programmes themselves – and should have been communicated at local and national levels, with *“95% of your job is wearing down the shoe leather on your shoes”* [Programme Manager 1]*.*

### Data and information systems

The appropriate IT systems have not been developed within the Irish health service and as a consequence IT systems overall are considered grossly out of date, which has impacted quite severely on the implementation of the NCPs, “*where the health system was already 10 years behind everywhere else in terms of IT, it basically got frozen and consequently its now 20 years behind everyone else”* [HSE Manager 4]*.* Information sharing was seen as critical to achieving implementation of NCPs, but the integration of information *“across primary, secondary and community care”* [Programme Manager 5] was difficult to achieve*.* The use of reliable, accurate, valid, complete and timely information in planning, operation and evaluation is a key feature of a modern health service [24]. The lack of IT systems also hampered the monitoring and evaluation functions available to the NCPs with “*one of our biggest challenges is that we cannot measure what we want to measure”* [HSE Manager 9]*.* Interventions and initiatives implemented could not be measured in terms of dose, reach and fidelity as this type of information is not routinely collected. Measuring performance can help develop an understanding of how well the health service is accomplishing goals and there is a need *“to have metrics so the performance metrics of the system are aligned with the designed models of care”* [HSE Manager 1]*.* This would allow for an analysis of where and what changes need to be made in order to improve the implementation of NCPs. Many of the respondents recognised the lack of national registers for common chronic diseases as a barrier to the advancement of patient care in general, and the NCPs in particular, stating that “*it’s essential that we have a national register for a range of conditions and it’s almost criminal that we don’t have them”* [Clinical Lead 5]*.*

### Changing the model of care

Certain events occurring generally within the Irish health service context were perceived by participants as forces that were outside the direct control of the NCPs, but nevertheless had serious impact on their implementation. For example, the contract negotiations between the State and general practitioners (GPs) was the most significant issue of this type *“and Programmes are going nowhere until you sort out the contract and the resourcing of primary care”* [GP Representative 1]. Some described GPs as *“missing partners”* [HSE Manager 4] in the early design of the NCPs, which resulted in them *“becoming more acute dominated than they were designed to be”* [HSE Manager 4]*.* This hampered subsequent attempts to redesign NCPs in an integrated manner across the health service. Recognition of the importance of the finalisation of the GP contract in the NCPs’ future sustainability was apparent,and its disruptive impact could not be overstated by participants *“that’s just the imperative of it”* [GP Representative 2] *.*

## Discussion

Attempts to change care within any health system is a complex intervention. The current study has successfully utilised the recent MRC guidance on conducting a process evaluation [10], and examined the barriers and facilitative factors to the implementation of a sub-set of the National Clinical Programmes within the Irish healthcare service. Basing intervention evaluation within the context of an evaluation framework allows researchers to understand which critical points an intervention needs to address and, after implementation, to identify why or why not an intervention worked in a specific context or setting [10]. Evaluating such a large system of change emphasises the relations between context, implementation and mechanisms. As the findings of the current study demonstrate, the NCPs comprised multiple interacting components. Themes central to the NCPs’ implementation included the organisational context in which the NCPs operate, leadership and clinical networks, and key barriers or facilitating factors relating to implementation, such as information technology, attitudes, resources, and forces outside of the NCPs thwarting change of the model of care.

### Leadership, governance and clinical networks

Organisational change efforts need clear and thoughtful consideration of their governance structure as much as the organisation’s operations do [25]. The governance of the NCPs centred on involving the professional Colleges in the nomination of Clinical Leads, thereby fostering buy-in and respect from fellow clinicians, the benefit of which was seen at the early stages of implementation of the NCPs. The programmes had, as a core part of their governance structure, the dyad of a Clinical Lead and a Programme Manager. All participants cited the importance of the Clinical Lead as being the key driver for change. Clinical Leads, despite a considerable workload, were dedicated, energetic and enthusiastic overall. The importance of leadership to facilitate organisational change and quality improvement was widely recognised by participants. Programme Managers, although not originally identified as ‘Leads’ per sé, have gained the recognition of their colleagues within NCPs that they are integral to the success of the programmes. Damschroder and colleagues, in the Consolidated Framework for Implementation Research, stress the importance of the formal appointment of leaders to act as champions [26]. This overall approach is also supported by Harvey and Kitson in their recent updating of the Promoting Action on Research Implementation in Health Services (PARiHS) framework [27].

Clinical networks have been established in many countries such as the UK [28, 29], France [30, 31], Canada [32], Australia [33, 34] and the US [35], and have shown success in breaching traditional professional boundaries, increasing compliance rates with evidence-based guidelines and have reported positive impacts on quality of care and patients outcomes [36]. These networks represent a shift away from the traditional hierarchical and bureaucratic systems of healthcare services, to one that fosters multidisciplinary collaboration, integration of services, and improved models of care where clinicians are engaged and committed to their development [33]. The NCPs’ foundation is based upon this clinical network model. The exact composition and typology of each clinical network depends on the purpose and the focus of the network (e.g., a disease construct such as cancer care or a network to improve the functionality of primary and secondary care across multiple conditions) [36]. Clinical networks within the NCPs included representation from clinical stakeholders such as physicians, and allied healthcare professionals such as nursing. Recently, there has been a move towards co-production of health services by including patients, families and even the general public within networks to design and reform the health service [37, 38]. The structure of the NCPs outlined allowed for inclusion of patients as part of the Clinical Advisory Group.

### Social and political context

The political climate can have a direct impact on the healthcare service, which may or may not work to the service’s advantage. The political nature of health service reform is therefore central to any understanding of a complex system [39]. In the current study, respondents spoke of the wider political and social context in which the NCPs operate. Respondents identified the pressure that the Minister of Health was under to tackle important but short term priorities like patients waiting on trolleys in emergency departments rather than the longer term ambition of the NCPs. This highlights the role that the media can play in influencing both the health policy agenda and health service delivery [40]. In Ireland, like in many other countries, healthcare provision is often in the national media, with attention usually riveted on the costs and quality of the care delivered or denied [41]. This wider social and political context made it difficult for the NCPs to focus on the more significant time intensive issues like the management of chronic disease, over headline making problems like waiting lists that dominate the media cycle. As a result of this political pressure the NCPs included, alongside the ‘triple aim’, an effort to reduce waiting lists and tackle trolley numbers. This demonstrates that large scale system change like the introduction and implementation of the NCPs should be contextualised in the wider socio-political environment [42].

### Resources

It was noted by participants that during the period of time known as the ‘Celtic Tiger’ years (mid-1990s to mid-2000s), which represented a period of rapid economic growth with increased spending in public services, there were not the improvements in health outcomes that may have been expected. The way services are funded is, therefore, an important consideration of integrated models [43]. There are two funding models currently under consideration within the Irish context – money follows the patient, and commissioning of services – which are discussed elsewhere in detail [44]. The NCPs started at a time in Ireland (2010), where the country was going into a deep and protracted economic recession, which led to a significant period of austerity in public spending [23]. Participants in the current study recognised that the resulting recruitment embargo, especially in terms of nursing staff, was particularly detrimental to the delivery of services. Despite the embargo now being lifted, participants reported continued problems with regard to having sufficient man-power to follow through on the operationalisation of some of the core business of the NCPs. It is therefore of increased importance that the Programmes incorporate workforce planning into their strategic plans for the future implementation and sustainability of the NCPs.

Participants in the current study noted that there were no financial incentives or rewards for improving service delivery within the NCPs, and that there was a sense of frustration that if one programme was making significant improvements in cost savings that these savings were not returned to that programme. Incentivisation appears entirely logical but the evidence base for its effectiveness is weak. For example, the evidence base for the UK Quality and Outcomes Framework remains patchy and inconclusive [45], and a recent Cochrane review found that the evidence base for implementation of financial incentives was not sound enough yet [46].

### Resistance to change

Much resistance to change can be avoided if effective change management is applied to the project from the very beginning and throughout [47]. Successful transformational change initiatives see the strategic value in communicating with people before, during and after a transition [48]. Participants in the current study consistently noted poor communication from the NCP central office within the HSE and vice versa. This lack of communication process led to unnecessary confusion within NCP networks. In order to overcome this problem in the future, there needs to be a clear and consistent communication plan. Strategies which delineate a clear vision of proposed changes help to promote participation in the change effort, rather than exclude staff from the change process [49]. The NCPs require such process in order to sustain and grow efforts. Policy makers should be reminded that incremental ‘bottom up’ reform, such as engagement with NCPs networks, may result in more effective and enduring effects on the health system than a ‘top-down’ approach which may alienate clinical and operational staff from the reform process [50], thus ensuring the sustainability of the NCPs into the future.

### Data and information systems

The current study has found that the NCPs are unable to generate or track changes or trends in areas of care that are within their remit. This seriously hampers the NCPs’ ability to know whether local or national interventions such as implementation of a new care pathway or a model of care are having the desired effect. The harnessing of technological advances and innovations, in particular within the realm of information and communication technologies, is an essential focus for health integration to achieve seamless care for patients [51]. A robust and system-wide information and communication technology system, that allows data management and patient tracking, is critical for effective chronic disease management [52, 53]. As long as the NCPs and the health system in Ireland in general are without an effective and efficient information technology structure, implementation of effective integrated care, management of chronic disease, and the NCPs themselves, will be seriously impeded.

### Changing the model of care

The NCPs set out to improve the performance of the healthcare system via the triple aim of improving quality, access and value [12]. Providing better care to individuals at a lower cost is not a new concept. The triple aims’ particular value is the advocacy for the inclusion of the population perspective in every healthcare improvement initiative [54]. An efficient and effective healthcare system means reorienting the model of care to one that prioritises primary and community services [55, 56]. This encompasses a shift from inpatient to outpatient and ambulatory care, and the move from curative to preventative care in order to place emphasis on using resources in the most effective and efficient settings, services and interventions [57]. Participants in the current study recognised the importance of primary care and general practice in particular. In Ireland, general practitioners are independent, autonomous practitioners who operate general practice services as sole traders or as partners within a larger practice. Many have contracts with the State to provide services to patients who are entitled to care under the General Medical Services [23]. Participants noted that while the on-going contractual issues were outside the direct sphere of influence of the NCPs, they nevertheless have significant impact on both the early development and current implementation of the programmes, especially in terms of the model of care. The successful completion of on-going complex contract negotiations between the HSE and general practitioners was recognised as a critical issue for the future sustainability of the NCPs.

### Study strengths and limitations

In this process evaluation we included a subset of the available NCPs (*N* = 7/33) and did not include all available NCPs. However, samples for qualitative studies are generally much smaller than those used in quantitative studies due to the aim of qualitative enquiry to gather rich, detailed information [58]. The subsets of NCPs chosen were from across acute and chronic health domains and participants represented key stakeholders involved in the NCPs, including Clinical Leads, Programme Managers and HSE management as well as GP, nursing and a patient representative. This study had a number of strengths relating to its methodology. In particular, a key strength was utilising the six stage process of analyses as it relates to qualitative thematic analyses [19]. Another strength of the study was the use of the Medical Research Council guidelines for process evaluation of complex interventions [10], which is an internationally recognised framework and informed the methodological approach taken in this research. This study also adhered to reporting guidelines for qualitative research by using a checklist for explicit and comprehensive reporting of qualitative research (COREQ) [17], which includes piloting the interview schedule, triangulation of analyses of the coding frame, data saturation and clarity of themes.

## Conclusion

Implementing change is challenging, especially large-scale health system change. By interviewing key organisational representatives who had developed and established a range of different NCPs we attempted to understand factors obstructing or facilitating the innovation and diffusion. There was support for change and improvement among the key stakeholders in the NCPs examined in this study. More formalised high-level political commitment to the initiative would be beneficial, as well as clarification of the executive authority lines in the NCPs. On-going training for the clinical leads in change management and clinical leadership, together with an agreed implementation strategy with appropriate incentivised funding, are needed at this stage of the process to take the NCPs to the next level. Finally, improved clinical information technology systems are required, both across the Irish healthcare systems as whole, and for the NCPs to measure outcomes.

## Appendix

### Sample Interview Schedule

1. What facilitative/helpful factors do you believe have assisted in implementation of the programme, and why?
  Prompts and follow on question if needed: To what extent are these factors generic (could be applied to other programmes) or context specific (only relevant to your programme)?. E.g., Enough resources, man-power, IT.
2. What barriers or blocks to implementation do you believe exist, and why?
  Prompts and follow on question if needed: (Things that have hindered the programme in some way?). Is there anything delaying or preventing implementation?
3. What do you see as the three most critical strategies required to achieve successful implementation across the HSE and nationally?
4. What would ‘full’ implementation look like, and where should the focus be?
  Prompts and follow on question if needed: In terms of the overall objectives of the programme, what needs to be done so that the model of care is in full operation?
5. Is there anything else that you would like to add?

Thank you for your time and willingness to participate.

## Abbreviations

COREQ: Consolidated Criteria for Reporting Qualitative Research
CSPD: Clinical Strategy and Programmes Directorate
HSE: Health Service Executive
MRC: Medical Research Council
NCPs: National Care Programmes
PARiHS: Promoting Action on Research Implementation in Health Services framework
WHO: World Health Organization
